# The Hospital

**Published:** 1917-11-17

**Authors:** 


THE
### INSTITUTIONAL WORKER
**Being a Special Supplement to "The Hospital"**

### OUR BUREAU OF INFORMATION.

### Rules for Correspondents.

1. Every letter must be accompanied by the coupon to be cut from the back cover (inside page) of The Hospital, current issue, and must contain the name and address of the correspondent with pseudonym for publication if desired. Replies by post cannot be given save under exceptional circumstances at the Editor's discretion.

2. Letters from Approved Homes in reply to special needs published in the Bureau should state terms and full particulars, and be sent prepaid under cover to the Editor of the Bureau with name written across coupon for identification.

3. Proprietors of Homes which have not yet been entered on the List of Approved Homes, but have spare accommodation likely to suit special needs, are invited to write for an application form for registration The fee for registration, which includes two announcements of the Home in the Bureau and other privileges, is 10s.

4. All communications to be addressed to the Editor of The Hospital, 28 Southampton Street, Strand, London, W.C. 2, and marked "Bureau of Information."

### Starting a Venereal Block.

### Answer to September Question.

By "Matron"

The question was :

It is proposed to start a venereal block, containing two wards of twelve beds each (male and female), a clinic, and an out-patients' department. (a) What nursing staff will be required, and how should it be distributed? (b) What equipment will be necessary for each department?

### The Nursing Staff.

This should consist of one sister, three fully trained staff nurses, two male nurses or orderlies. The sister to be in charge of all three departments, with two nurses and one orderly on day duty and one nurse and one orderly for night duty.

The sister would attend the clinic and out-patients' departments, having a nurse to help her with the female section and an orderly for the male section. This would leave one nurse in charge of the wards. The clinic and out-patients' department will probably take up two or three hours on three or four days of the week, during the afternoon, when the work of the wards is at its highest, when one nurse should be able to manage. Off-duty could be given in the mornings and evenings, and half-days on those days when there are no clinic or out patients to attend to.

During the night one nurse will be in charge, with an orderly under her. I specify trained nurses preferably to nurses still in training, taking into consideration that this branch of nursing requires that those who undertake it must fully realise the nature of the work they are engaged upon, and should by this time understand the full value and meaning of asepsis and how to guard against infection.

There are often dressings and treatment to be done for the male patients, which renders it essential to have either male nurses or orderlies.

If untrained men are employed, they, of course, will have to be taught what is required of them. My own experience is that ex-soldiers often make good orderlies and are very teachable.

### Equipment.

It is assumed that bedsteads, lockers, and other ward furniture are already at hand.

Bed-Linen.

18 white quilts.
96 white blankets.
18 red blankets.
96 large sheets (linen).
120 draw sheets (unbleached grey twill).
96 pillow-cases (linen).
18 calico mattress-covers.
12 red rubber sheets (2 yards long).
6 red rubber sheets (draw).

Each mattress to be covered with long rubber sheet, and draw rubber sheet used only when necessary. Red blankets to take the place of quilts during the night.

Linen for Patients, etc.

60 cotton night shirts and gowns.
12 flannel night shirts and gowns.
48 bed jackets.
60 patients' towels
48 doctors' and sisters' hand towels.
24 roller towels.
24 tea towels.
48 dressing towels (1 yard square, calico).
12 hot-water bottle covers.
24 lavatory or bowl cloths.
6 tablecloths.
60 locker cloths (serviettes).
6 dressing-gowns.
24 surgeons' coats.
4 soiled-linen bags.

It is customary to have all soiled linen disinfected before sending to the hospital laundry from this department. As constant disinfecting has a somewhat deteriorating effect on linen, it might be advisable (if possible) to stock this department from other wards' linen, replacing same with new stock.

The surgeons' coats, roller, hand, and dressing towels and bowl cloths will supply all three departments.

Kitchen Equipment.

30 cups and saucers.
30 meat, pudding, and small plates.
24 large knives (metal handles, one piece).
24 large forks (Nevada silver).
24 dessert spoons (Nevada silver).
24 teaspoons (Nevada silver).
4 serving spoons (enamelled).
1 carving knife and fork.
1 bread knife.
2 enamelled teapots.
2 enamelled jugs.
24 enamelled egg-cups.
12 soup bowls.
4 pepper castors.
4 salt cellars.
4 mustard pots.

One large block-tin fish-kettle, to allow of crockery and cutlery to be boiled each day if so required.

Sanitary Block.

4 round enamelled bedpans.
2 enamelled bedpans, slippers.
1 enamelled douche receiver.
6 enamelled male urinals.
2 enamelled urine measure jugs.
1 enamelled mop jar.
1 enamelled soiled-dressing pail.
1 urine test stand.
6 urine specimen glasses.

Bathroom.

12 enamelled washing bowls.
12 enamelled dressing bowls (large).
12 enamelled dressing bowls (small).
9 enamelled kidney receivers.
3 enamelled square receivers.
3 enamelled catheter trays.
2 enamelled douche cans.
2 enamelled or copper sterilisers.
3 dressing drums.
4 Higginson syringes.
4 clinical thermometers.
1 bath thermometer.
6 medicine measures.
6 dressing mackintoshes.
12 hot-water bottles.
6 enamelled expectoration cups.

Rubber gloves, instruments, and other surgical appliances will come under the medical officer's control.

### Questions for November.

**1. The Equipment of an Out-Patient Department.**

State what you consider necessary for the general equipment of a new Out-Patient Department and Accident Room, and the procedure to be adopted for the provision of such requirements.

**2. How to Organise a Mothers' and Babies' Welcome.**

Outline a simple scheme which a midwife working independently and single-handed in a scattered country district could adopt to start a "Mothers' and Babies' Welcome."

### RULES.

The following rules must be observed:—

1. Contributions must be written on one side of the paper. Brevity and terseness are desirable features. The MSS. must bear the name and address of the sender and be accompanied by coupon to be cut from the back cover (inside page) of the current issue of THE HOSPITAL. A pseudonym must be chosen if the name is not to be published.

2. Contributions must be addressed to the Editor of THE HOSPITAL, Institutional Worker Supplement, 28 & 29 Southampton Street, Strand, London, W.C. 2, should reach him before the end of the current month, and be marked in the left-hand corner "Facts and Figures."

**A minimum payment of Half-a-Guinea will be made for each published answer.**

### QUESTIONS INVITED.

IN connection with our Question Box, we offer every month five shillings each for the two best questions which are sent in for consideration. The questions may be on any institutional subject and concern any department, from the secretary's to the porter's, or the matron's to the domestic staff; the one essential is that they must be practical and deal with points that have a definite relation to hospital or institutional administration, upkeep, finance, or management. Points relating to artificers' work, housekeeping, the laundry, out-patients, and other practical matters will be welcome. It is hoped that thus, when difficult or doubtful points arise in the course of their work, institutional workers will be encouraged to put them in the form of a question and send them to the Editor, THE HOSPITAL Bureau of Information, 28 Southampton Street, Strand, W.C. 2, marked "Question Box."

The best questions will be published in due course and our readers will be given the opportunity of answering them. Thus all institutional workers may be able to co-operate with the view of helping the work and smoothing the difficulties of each other. In short, we want the Question Box of the *Institutional Worker* to be freely used by every worker habitually.

### ENQUIRIES AND ANSWERS.

### *WAR MATTERS.*

**Women's Auxiliary Corps.**

YOU are not eligible for enrolment in the Women's Army Auxiliary Corps without a letter from the head of your department testifying that your services in your present position can be dispensed with.—V. A. D.

**Deaf Discharged Soldiers.**

A SPECIAL Aural Board has been set up by the Pensions Minister at the Central Nose, Throat, and Ear Hospital, to deal with cases of deaf discharged soldiers. We believe that the classes for instruction in lip reading are still being held at the College of the National Association for the Oral Instruction of the Deaf at 11 Fitzroy Square, London, with morning and afternoon instruction. Write to the Chairman of the Aural Board without delay, as it is undesirable to lose so much time in a case such as yours.—DISCHARGED SOLDIER.

**Training the Blind at St. Dunstan's.**

AMONGST the occupations in which training is given to the Blind at St. Dunstan's, Regent's Park, London, N.W., are carpentry, boot-repairing, mat-making, basket-making, and telegraphy. Massage is taught in the Massage Department at the National Institute for the Blind, and the course is that prescribed by the Incorporated Society of Trained Masseuses. The men are also given instruction in poultry-farming, market-gardening, and way-finding. Braille reading, writing, typewriting, and shorthand writing by the Braille system are other industries in which instruction is given.—INTERESTED NORTHERNER.

### *MISCELLANEOUS.*

**Magnetic Wave Treatment.**

THE "Bachelet" Magnetic Wave treatment to which you refer is provided by a generator of American design. It is said to be of some value in the treatment of nervous conditions. The current generated resembles somewhat the Morton Wave Current delivered by a static machine. The particular apparatus used is, however, considerably smaller than other devices used for generating the magnetic field in which to place the patient. It comprises two generators; one is placed in front and the other behind the patient and some distance from him. As soon as the generators are brought into action lines of force travel from one to the other, the patient's body being in the path of a current which reaches in volume over 3,000 milliamperes. It is said that this does not unduly excite the nervous system, but, on the contrary, acts as a nerve sedative.—H. M. T.

**Preliminary Air-Raid Warning.**

ARRANGEMENTS have been made with Scotland Yard to issue to the hospitals a preliminary warning when the coast defences are first aware of the approach of enemy aircraft. This is in addition to the intermediate and air-raid action warning. Whether or not you decide to ask the authorities to send you this warning must depend upon the length of time you require for the needful preparation at your hospital for the safety of the patients and the treatment of casualties. If you take notice of this warning you will, of course, find that in many instances your preparations are to no purpose. The question is one which your circumstances must help you to decide; you must remember, however, that if the aircraft penetrate the defences very little time elapses between the preliminary and final warnings.—SMALL LONDON HOSPITAL.

### *EMPLOYMENT AND TRAINING.*

**Royal College of Surgeons in Ireland.**

THE fee for candidates holding a surgical diploma other than that of the Royal College of Surgeons in Ireland is £21; Diploma in State Medicine, £10 10s.; Conjoint Examinations of the Royal College of Physicians and Royal College of Surgeons in Medicine, Surgery, and Midwifery, £42. The address of the College is Stephen's Green West, Dublin.—PAT.

**Training in Ipswich.**

PROBATIONERS are given a four years' course of training, including massage, at the East Suffolk and Ipswich Hospital, Ipswich. Application should be made to the Matron.—C. O. F.

### *THE HOUSEKEEPERS' DEPARTMENT.*

**Lemon Drop Cakes.**

To make thirty-six of these drop cakes you will require 2 oz. of butter, margarine, or lard, 1 good teaspoonful of egg-powder, ½ teaspoonful mixed spice, ½ teaspoonful carbonate of soda, 2 tablespoonfuls of milk, 3 tablespoonfuls of golden syrup or treacle, and essence of lemon to taste. Rub the fat to a cream, mix the flour and spice, which add to the fat, together with the syrup; then dissolve the soda in the milk and mix all well together, after putting in the essence drops. Put the mixture into well-greased patty pans and bake for half to three-quarters of an hour.—NO SUGAR.

**Recipe for Lemon Syrup Pudding.**

To make a fairly small-sized pudding, sufficient for about six people, you will require 2 oz. bread-crumbs. 2 oz. flour, 3 oz. suet, rind and juice of 1 lemon, 1 teaspoonful egg-powder, ½ teacupful of milk, and 2 tablespoonfuls of syrup. Chop the suet and lemon rind very fine, and mix them with the flour and bread-crumbs and egg-powder; then add the lemon juice and syrup; last of all, put in the milk. Beat all the ingredients well together, and steam in a well-greased basin for two hours.—AVIS.

